# Optic tectal superficial interneurons detect motion in larval zebrafish

**DOI:** 10.1007/s13238-018-0587-7

**Published:** 2018-11-12

**Authors:** Chen Yin, Xiaoquan Li, Jiulin Du

**Affiliations:** 1 Institute of Neuroscience, State Key Laboratory of Neuroscience, Center for Excellence in Brain Science and Intelligence Technology, Chinese Academy of Sciences, Shanghai, 200031 China; 2 School of Future Technology, University of Chinese Academy of Sciences, Beijing, 100049 China; 3 School of Life Science and Technology, ShanghaiTech University, Shanghai, 200031 China

**Keywords:** optic tectum, motion detection, direction selectivity, visual adaptation, zebrafish

## Abstract

Detection of moving objects is an essential skill for animals to hunt prey, recognize conspecifics and avoid predators. The zebrafish, as a vertebrate model, primarily uses its elaborate visual system to distinguish moving objects against background scenes. The optic tectum (OT) receives and integrates inputs from various types of retinal ganglion cells (RGCs), including direction-selective (DS) RGCs and size-selective RGCs, and is required for both prey capture and predator avoidance. However, it remains largely unknown how motion information is processed within the OT. Here we performed *in vivo* whole-cell recording and calcium imaging to investigate the role of superficial interneurons (SINs), a specific type of optic tectal neurons, in motion detection of larval zebrafish. SINs mainly receive excitatory synaptic inputs, exhibit transient ON- or OFF-type of responses evoked by light flashes, and possess a large receptive field (RF). One fifth of SINs are DS and classified into two subsets with separate preferred directions. Furthermore, SINs show size-dependent responses to moving dots. They are efficiently activated by moving objects but not static ones, capable of showing sustained responses to moving objects and having less visual adaptation than periventricular neurons (PVNs), the principal tectal cells. Behaviorally, ablation of SINs impairs prey capture, which requires local motion detection, but not global looming-evoked escape. Finally, starvation enhances the gain of SINs’ motion responses while maintaining their size tuning and DS. These results indicate that SINs serve as a motion detector for sensing and localizing sized moving objects in the visual field.

**Electronic supplementary material:**

The online version of this article (10.1007/s13238-018-0587-7) contains supplementary material, which is available to authorized users.

## INTRODUCTION

Animals distinguish prey, conspecifics and predators from the constantly changing world through combing a number of objective features, among which motion is an essential one (Mauss et al., 2017). In visual areas of the brain, local circuits integrate visual inputs to represent motion information. The optic tectum (OT), the visual center in low vertebrates and the homolog of the superior colliculus in mammals, is the primary target of RGC axon terminals and transmits behavior-relevant information down to motor outputs, which orient the body axis toward or away from perceived objects (Dunn et al., 2016; Gahtan et al., 2005; Nevin et al., 2010). Thus, the processing of motion information within the OT is instrumental to object monitoring and visuomotor transformation. With regard to motion detection, an important feature that can be extracted is the direction of a moving object. Different cell types within the retina have been characterized to be tuned to motion directions (Barlow and Hill, 1963; Kim et al., 2008; Oyster and Barlow, 1967; Vaney et al., 2012; Wyatt and Daw, 1975). In zebrafish, different subtypes of DS-RGCs and orientation-selective (OS) RGCs were also identified (Nikolaou et al., 2012). Beyond the retina, downstream DS cells, especially distinct neuronal subtypes within the OT are just beginning to be characterized (Grama and Engert, 2012; Hunter et al., 2013; Niell and Smith, 2005). Specifically, two subtypes of GABAergic DS cells were identified with a matching laminar distribution of DS-RGC inputs (Gabriel et al., 2012). In addition to directional information, RGCs also encode size information for moving objects, endowing the animal the ability to localize and distinguish sized local objects (Preuss et al., 2014). Ethologically, motion information provides essential cues to signal physiological meaning such as food resources and threatening and thus is under elaborate processing (Borst and Euler, 2011).

Resided in the input layer of the tectal neuropil, superficial interneurons (SINs), a population of GABAergic interneurons, were reported to be involved in prey capture (Del Bene et al., 2010). However, the functional properties of SINs specialized for specific visuomotor behaviors remain poorly understood. Here, we address this question by using *in vivo* whole-cell recording and functional calcium imaging in larval zebrafish. We identified two subsets of DS SINs with preferred directions separated by ∼120° and approximately cover rostral to caudal (RC) directional information separated by ∼120° SINs exhibit sustained high frequency firing with current injection, transient ON- and OFF-type light responses, large RF consistent with their broadly stratified arborization, and mainly receive excitatory inputs. SINs show size-dependent responses to moving dots. Further characterization reveals that SINs are activated by a moving object but not a static one and capable of showing sustained responses to a moving object within the RF, which could be explained by less visual adaptation to paired-pulse stimuli than that of periventricular neurons (PVNs). Behaviorally, ablation of SINs impairs prey capture which requires fine local motion detection but not global looming-evoked fast escape. In addition, motion responses of SINs show gain modulation by feeding state, in which starvation increases response amplitude and ratio of responsive cells while maintaining size tuning and DS properties.

## RESULTS

### Electrophysiological properties of SINs

**Figure 1:**
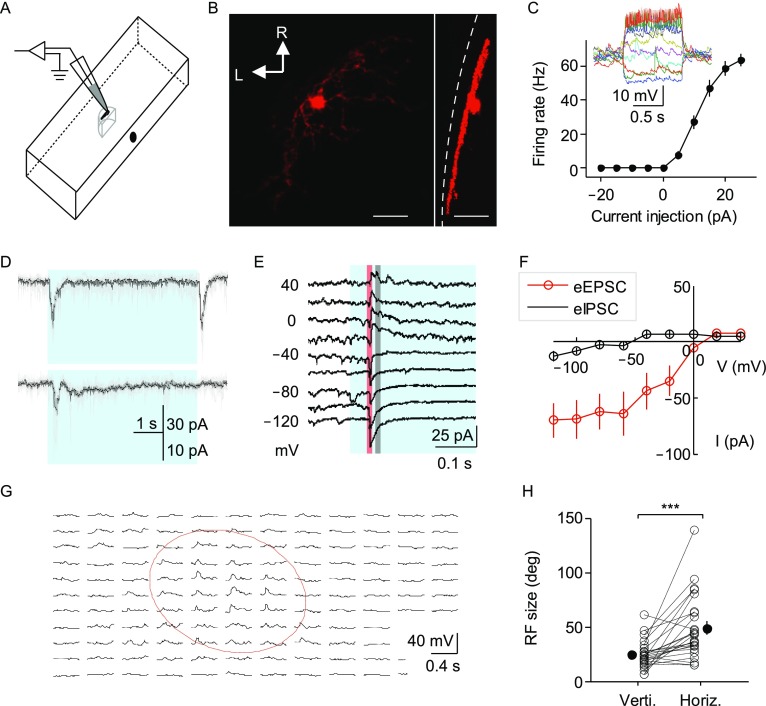
**Electrophysiological characterization of SINs**. (A) Simplified schematic showing recording paradigm. Black dot indicates visual stimulation on the screen. (B) *In vivo* confocal image of single photo-converted SIN. Left: top view; right: side view (77° rotation of image stack). Dashed line indicates location of skin above the surface of the tectum. (C) Summary of current-frequency relationship (*n* = 56). Inset: voltage traces with current injection. In this and subsequent figures, error bars indicate SEM. (D) Current traces showing transient ON/OFF and OFF responses to whole-field stimulation. In this and subsequent figures, blue area, gray and black traces indicate stimulation window, individual representations and averaged responses, respectively, unless otherwise mentioned. (E) Current traces with holding potentials from −120 mV to 40 mV in 20 mV step. (F) Summary of the I–V relationship (*n* = 9). (G) Spatial RF (outlined by dashed line) of one SIN. (H) Summary of RF sizes (*n* = 25; *P* = 1.4 × 10^−4^, Wilcoxon signed-rank test). Scale bar, 20 μm

We first performed *in vivo* whole-cell recording in 7–8 dpf Tg(Gal4-1156t, UAS:Kaede) larvae with a custom-built recording chamber. Recorded SINs were from the tectal neuropil contralateral to the visually stimulated eye (Fig. 1A). The morphology of a representative SIN photo-converted (405 nm laser, 1 s) from a dark reared Tg(Gal4-1156t,UAS:Kaede) larva is illustrated in Fig. 1B. SINs extend broadly stratified arborization at the superficial layer of the tectal neuropil, where PVNs receive and relay visual signals to downstream cells within the tectum. SINs showed sustained non-adapting high frequency firing upon super-threshold current injection (Figs. 1C, S1A and S1B), reminiscent of the fast spiking interneurons that control sensory responses and information flow in rodents (Cardin et al., 2009).

To directly investigate SIN visual functions, whole-field dimming stimulation was imposed to the larva. The majority of SINs showed transient ON and OFF responses (84%; *n* = 77/92) while the rest showed only transient OFF responses (16%; *n* = 15/92; Fig. 1D). To compare excitatory and inhibitory inputs, diming stimulation evoked responses at different holding potentials were temporally divided into two synaptic components after stimulation onset (Fig. 1E). The early component (42 ± 1 ms to 82 ± 10 ms after stimulation onset) was large and reversed at ~0 mV, suggesting strong excitatory inputs from RGCs. Meanwhile, the late component (127 ± 24 ms to 168 ± 32 ms after stimulation onset) was weak and reversed at ~−60 mV, equal to the reversal potential of chloride currents(~−60 mV), suggesting weak or absence of inhibitory inputs (Fig. 1F). We calculated evoked conductance from linear regression over a fixed voltage range from −80 to −20 mV to avoid the sublinear behavior at the extremes of the I–V relationship. The ratio of excitatory conductance (0.51 ± 0.09 nS) to inhibitory conductance (0.2 ± 0.03 nS) was 2.6, suggesting inputs to SINs are dominated by barrages of excitation. We next mapped the RF properties of SINs, which reflect the spatial arrangement of inputs and support fundamental visual functions (Figs. 1G, S1C and S1D). The RF size was quantified as the average of the half width at half maximal from the two axis of the ellipse that was fitted from a 2D Gaussian model. SINs had large spatial RF size of 37° ± 3° in average. In addition, we found that the averaged RF size in the horizontal axis (49° ± 6°) is significantly larger than that in the vertical axis (25° ± 3°; Fig. 1H), which reflects more coverage and intense information processing of the horizontal field.

### A proportion of SINs are direction selective

**Figure 2:**
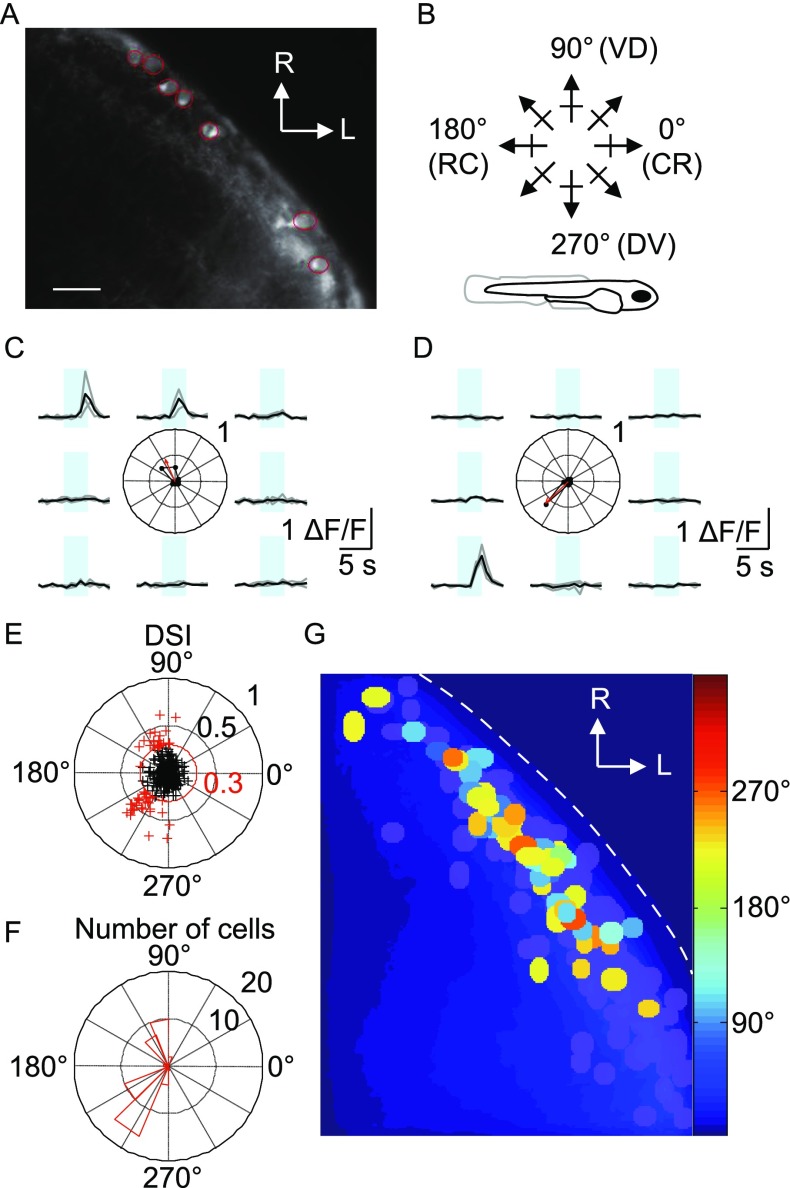
**Direction selectivity of SINs**. (A) Fluorescent signal in Gal4s1156t, UAS:GCaMP-HS larva. Region of interests (ROIs) are demarcated by red lines. (B) Schematic showing direction of motion of bars relative to fish body axis (Bottom). VD: ventral to dorsal. DV: dorsal to ventral. (C and D) Ca^2+^ transients from two somata. Center: polar plot of normalized peak amplitudes of Ca^2+^ transients. Arrow shows vector sum of normalized peak amplitudes, indicating PD and DSI. (E) DSI and PD for all somata. Red circle marks a DSI of 0.3, used as criterion for DS. (F) Histogram of PDs for all DS cells from (E) in red. (G) All responsive somata from grouped experiments, color-coded according to PD. Gray somata showing responsive but not DS cells (total number of cells imaged: 304; responsive cells: 247, 81.3%). Scale bar, 20 μm

To understand whether SINs are tuned to directional motion, we measured DS of SINs with black moving bars. In order to identify DS SINs with population imaging, we expressed GCaMP-HS, an improved version of GCaMP with a higher refolding activity and sensitivity to the change of cellular Ca^2+^ concentrations (Muto et al., 2011), in SINs (Fig. 2A). The moving bar was in eight directions evenly spanning 360° (0° corresponds to caudal to rostral [CR] direction; Fig. 2B). We calculated the preferred direction (PD) and direction selectivity index (DSI) of all responsive neurons and revealed that a proportion of SINs (52 of 247 = 21.1% of responsive cells) were DS (Fig. 2C and 2D). Rather than having a single PD or uniformly distributed, the distribution of PDs revealed two populations of DS SINs centered at 112° and 233°, which in collection approximately fill the caudal hemisphere of motion directions (90°–270°; Fig. 2E and 2F). A composite color-coded stack of all DS SINs from all experiments were projected onto the averaged tectal fluorescence image that was aligned using the surface boundary as a reference (Fig. 2G). The identified two populations were distinguished by coded colors and not clustered but intermingled at the superficial neuropil layer. In addition, we also calculated the OS of these SINs. Correspondently, OS SINs were also intermingled (Fig. S2).

### SINs are size-tuned to moving objects

**Figure 3:**
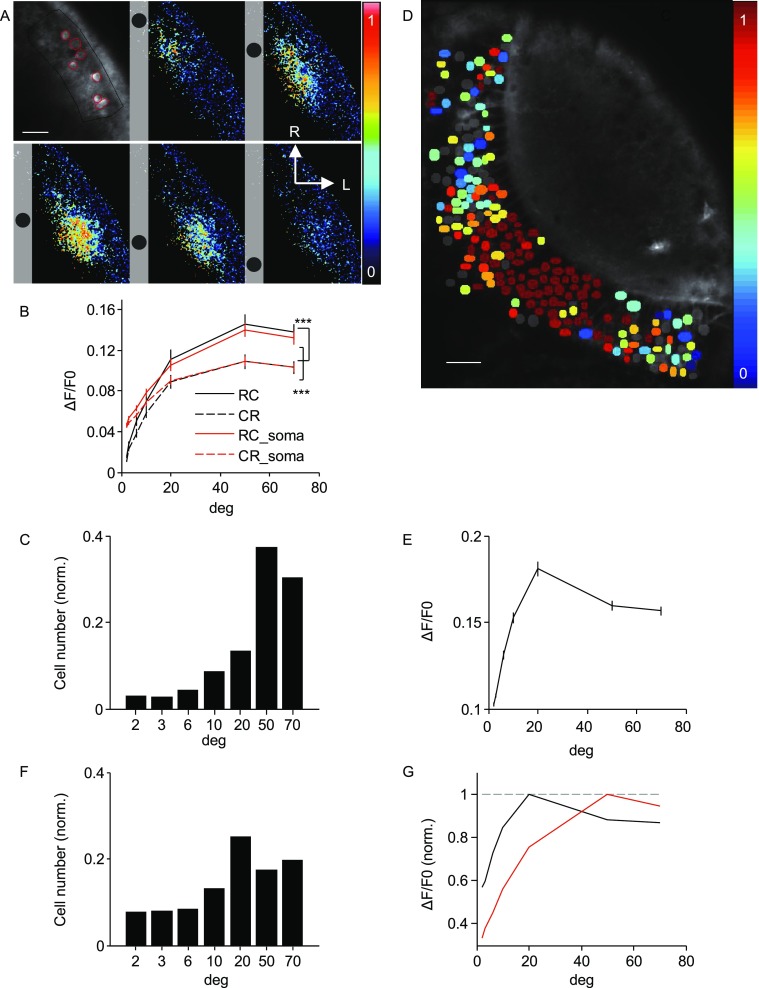
**Size tuning of SINs**. (A) Montage showing responses of SINs to moving dot stimulus of 10°, color-coded according to normalized responses. Relative position in each panel indicates direction of motion. Top left, fluorescence image showing analyzed ROIs demarcated by lines (population in black; somata in red). (B) Summary of responses to moving dots from population (*n* = 47 fishes, *P* = 2.3 × 10^−10^, two-way ANOVA) and responsive somata (*n* = 254/338, *P* = 1.8 × 10^−9^, two-way ANOVA). (C) Size preference distribution for all somata (*n* = 254/338 cells from 47 fishes). (D) Responses of PVNs to moving dot stimulus of 20°, color-coded according to normalized responses. Gray somata showing nonresponsive cells. (E) Summary of responses to moving dots from all somata (*n* = 1,651). (F) Size preference distribution from all somata. (G) Comparison of tuning curves between SINs (red) and PVNs (black)

Apart from moving direction, information about the size of an object is critical for behavioral choices (Bianco et al., 2011; Trivedi and Bollmann, 2013). To further investigate SIN size tuning property, we performed imaging and whole-cell recording experiments using moving dots that are more behaviorally relevant than bars. Moving dot evoked robust Ca^2+^ transients (Fig. 3A). Consistent with the result with moving bar stimulation (Del Bene et al., 2010), SINs are tuned to large stimuli (Fig. 3B). Responses in RC direction were significantly larger than CR direction, consistent with the combined PD of SINs. In addition, we found that Ca^2+^ transients reached a plateau at ~ 50°, indicating a maximal excitation at ~ 50° that is consistent with SIN’s average RF size (Fig. 1H). The distribution of preferred sizes of individual SINs revealed a maximal preference of 50 degree and an overall large size preference (Fig. 3C). Consistently, whole-cell recordings revealed that SINs received larger excitatory postsynaptic currents with dots of increasing size passing through the RF (Fig. S3).

Then we asked how PVNs, the potential downstream tectal cells, respond to moving dots with different sizes. To this end, we measured the size tuning property of PVNs with Tg(HuC:GCaMP5) line, which expresses GCaMP5 pan-neuronally (Fig. 3D). The distribution of preferred sizes of individual PVNs revealed a maximal preference of 20 degree (Fig. 3E and 3F). A direct comparison of the tuning curves between SINs and PVNs revealed sharpened tuning for PVNs, in which local SIN inhibitory input is likely to play a role (Fig. 3G).

### SINs detect and show sustained responses to moving objects

**Figure 4:**
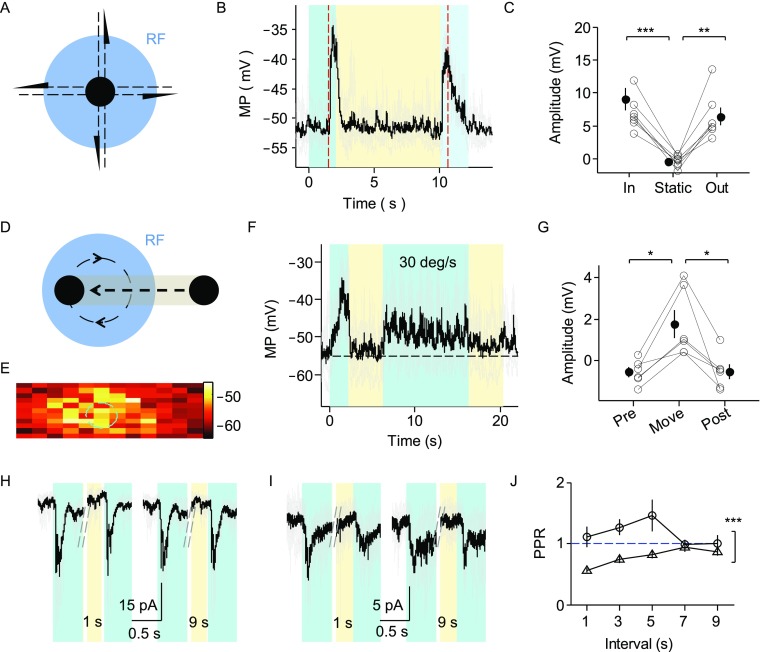
**Motion response and visual adaptation of SINs**. (A) Schematic showing moving dot stimulation. Dashed arrows indicate moving directions. (B and C) Voltage traces (B) and summary of data (C) (*n* = 7; moving in versus static: *P* = 2.2 × 10^−4^; moving out versus static: *P* = 0.0018; both Student’s *t*-test) for responses to one dot in moving and static. Red dashed lines in (B) indicate dot moving into or out of the RF. Response peaks of moving in and out period were compared to averaged membrane potential from 1 s after static period onset and 1 s before static period offset, respectively (C). (D) Schematic showing moving dot stimulation. Dashed arrows indicate moving directions. (E) Heat map of the RF. Blue dashed line demarcating the rotating area. (F and G) Voltage traces (F) and summary of data (G) (*n* = 6; moving versus pre-static: *P* = 0.0313; moving versus post-static: *P* = 0.0313; both Wilcoxon signed-rank test) for responses to dot in rotating in 30°/s and static. (H and I) Current traces for responses to paired visual stimuli for one representative SIN (H) and PVN (I), respectively. (J) Comparison of visual adaptation between SINs (*n* = 8; circle) and PVNs (*n* = 9; triangle) (*P* = 1.6 × 10^−4^, two-way ANOVA)

To study whether SINs only respond to an object in moving or both in moving and static state, we designed the following three-phase stimulation protocol: a dot in 10° moving from outside of the RF into the center, static for 8 s, and then moving out of the RF along the same direction (Fig. 4A). We found that the dot only evoked response when it was moving but not static (Fig. 4B). From summed data averaged from all four directions as they all evoked robust responses for individual cells (Figs. 4C and S4A), we conclude that SINs are activated by moving objects. Furthermore, to study whether SINs keep the moving information, we designed the following four-phase stimulation protocol: a dot in 10° moving from outside of the RF into the RF near the center, static for 4 s, rotating within the RF for 10 s, static for 4 s, and then offset (Fig. 4D and 4E). Except for evoked response during moving-in phase as the previous stimulation paradigm (Fig. 4B), the dot evoked sustained responses while it was rotating within the RF at a series of moving speeds (Figs. 4F and S4B). From summed data of response amplitude calculated from mean membrane potential within rotating period subtracting a pre-stimulus baseline, we conclude that SINs show sustained responses to moving objects within the RF independent of moving speeds (Figs. 4G and S4C). To further understand the mechanism underlying the sustained responses, we compared the visual adaptation property of SINs to PVNs. To this end, pairs of dimming stimuli were imposed, from which off responses were calculated. Notably, the second dimming pulse evoked as large currents as the first pulse for SINs even at the minimal interval, while PVNs only gradually recovered the second response (Fig. 4H and 4I). Collectively, SINs recovered evoked response with paired-pulse ratio (PPR) reaching 1 within 1 s. In contrast, PPR of PVNs recovered to 1 much slower with a power fitted time of 13.4 s (Figs. 4J and S4D). Thus the result that SINs are less adapted to visual stimulation suggests that SINs are well suited to capturing moving information.

### SINs are crucial for prey capture but not escape

**Figure 5:**
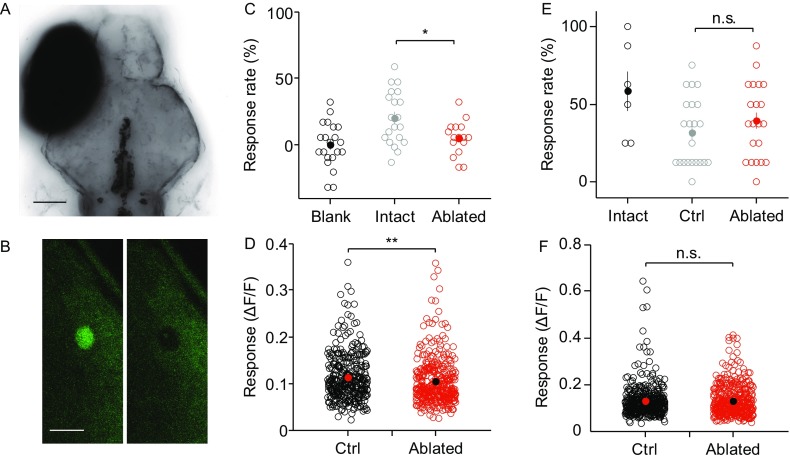
**SINs are crucial for prey capture but not looming-evoked escape**. (A) Confocal image of a 7 dpf larva with ipsilateral eye removed. Scale bar, 100 μm. (B) SIN before (Left panel) and after (Right panel) laser ablation. Soma is destroyed and cell debris is visible. Scale bar, 10 μm. (C) Prey capture reduced in SIN ablated larvae (*n* = 16) relative to control (*n* = 20, *P* = 0.0134, Student’s *t*-test). Blank indicates no fish control. (D) Prey-like moving dot stimulation (3°) evoked response in PVNs reduced in SIN ablated larvae (*n* = 348 from 13 larvae) relative to control (*n* = 362 from 15 larvae, *P* = 0.0011, Wilcoxon rank-sum test). (E) Looming evoked escape unaffected in SIN ablated larvae (*n* = 21) relative to control (*n* = 22, *P* = 0.3132, Wilcoxon rank-sum test). Intact fish serve as control. (F) Looming evoked tectal responses unaffected in SIN ablated larvae (*n* = 392 from 6 larvae) relative to control (*n* = 414 from 7 larvae, *P* = 0.9004, Wilcoxon rank-sum test)

Feeding is essential for survival, which is primarily visually guided and requires the optic tectum in zebrafish (Gahtan et al., 2005). Correspondingly, we suppose that the behavioral implication of SIN direction and size tuning is the ability to recognize edible objects during prey capture, which requires detection of fine motion objects. In order to measure how SINs affect motion related behaviors, we firstly removed the ipsilateral eye as the close proximity of SINs to eyes interferes photo-ablation manipulation (Fig. 5A). This also provided convenience for ipsilateral SIN manipulation as only the contralateral eye remained intact. We ablated ipsilateral SINs with two photon illumination in Tg(Gal4-1156t, UAS:Kaede) transgenic larvae (Fig. 5B). We found that fish with ipsilateral SIN ablation consumed significantly fewer paramecia than unablated control animals (Fig. 5C). Accordingly, prey-like stimulation (3° moving dot) evoked significantly smaller responses of PVNs in ablated than in control animals (Fig. 5D). Furthermore, we compared the global looming stimulation evoked fast escape between these animals to test whether SINs generally affect motion involved prey capture and avoidance behaviors. The result showed that SIN ablation did not impair looming evoked avoidance (Fig. 5E). Accordingly, no difference was observed in looming evoked tectal responses between SIN ablated and control animals (Fig. 5F). As a control, we found stationary dimming evoked escape and locomotion ability were unaffected by SIN ablation (Fig. S5).

### Brain state-dependent gain modulation of motion detection of SINs

**Figure 6:**
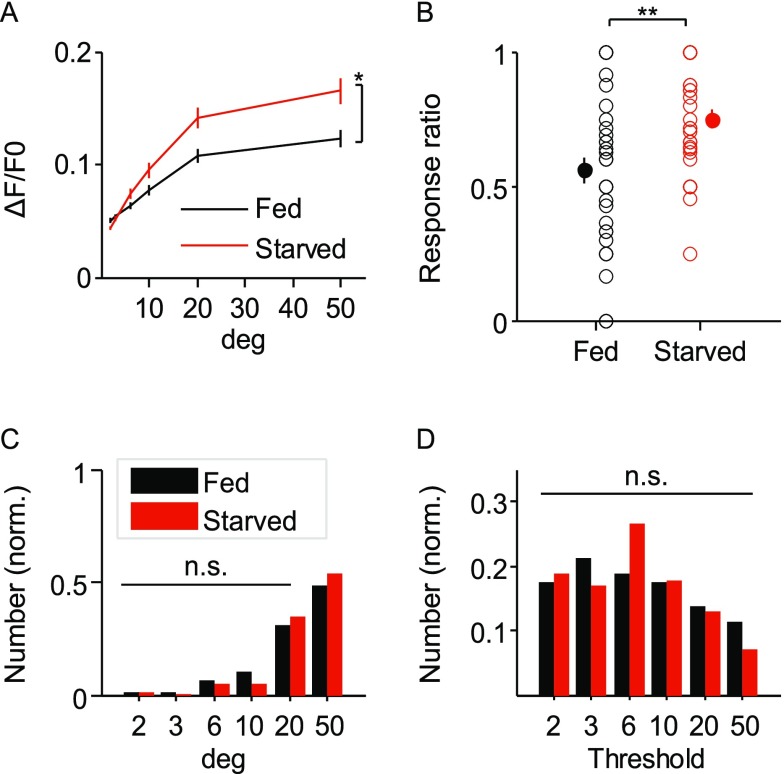
**Gain modulation of SIN motion responses by brain state**. (A) Summary of responses to moving dots from responsive somata between fed (*n* = 132/247) and starved (*n* = 170/231) larvae (*P* = 0.0109, two-way ANOVA). (B) Summary of ratio of responsive neurons between fed (*n* = 39 fishes) and starved (*n* = 39 fishes) larvae (*P* = 0.0024, Student’s *t*-test). (C) Distribution of tuned sizes remained unchanged in starved larvae (*n* = 170/231) relative to fed control (*n* = 132/247, *P* = 0.5246, Kolmogorov-Smirnov test). (D) Distribution of response sensitivity remained unchanged in starved larvae (*n* = 170/231) relative to fed control (*n* = 132/247, *P* = 0.9909, Kolmogorov-Smirnov test)

To explore whether SIN visual motion processing is modulated by feeding state, we firstly compared the size tuning property between fed and food-deprived larvae using moving dot stimuli. While SINs still showed size-tuned responses to moving dots, the response amplitudes were larger in starved than in fed larvae, indicating a gain modulation of motion responses by starvation (Fig. 6A). Furthermore, we compared the ratio of responsive SINs in individual larvae. Starved larvae showed significantly higher ratio of responsive SINs than fed larvae, indicating that more SINs in the available SIN pool were recruited in visual motion processing under starved state (Fig. 6B). Thus, the feeding state-dependent output of SINs emerges by a dual mechanism that comprises of both the increased responses of individual SINs and the recruitment of a larger pool of responsive SINs. In order to test whether starvation changed the size preference and response sensitivity of SINs, we compared their tuned sizes and response thresholds. The histogram distribution revealed neither difference in tuned sizes nor response thresholds, indicating unaffected response pattern by starvation (Fig. 6C and 6D). In parallel, a similar gain modulation by starvation to direction tuning was also observed between fed and food-deprived larvae (Fig. S6), which, together with results on rodents (Fu et al., 2014; Lee et al., 2014; Niell and Stryker, 2010; Polack et al., 2013), reveal a general role of gain modulation by brain states.

## DISCUSSION

In the present study, we provide direct evidence for motion detection properties of SINs within the tectum. As SINs are located at the most superficial neuropil layer where RGC afferents terminate, it is probable that SINs themselves receive only RGC excitatory inputs. While the negligible inhibitory inputs evoked by dimming stimulation may result from potential mutual inhibition among SINs.

By combining the merits of GCaMP-HS, an improved version of calcium indicator, and behaviorally relevant visual stimuli, our results demonstrate that SINs respond robustly to 10° moving bars and moving dots of various sizes. The observation of the tuning properties of the two populations of DS SINs is supported by the result from OGB-labeled superficial neuropil cells (Hunter et al., 2013), although a minor population of SINs that prefer caudal-to-rostral motion were reported (Abbas et al., 2017). SIN’s DS may directly derive from the subset of DS-RGCs, thus enabling SINs specialized for detecting motion along the horizontal axis of the visual field. The stronger RC inhibition derived from SINs, together with relatively strong CR-tuned retinal input to the tectum (Maximov et al., 2005; Nikolaou et al., 2012), may contribute to the prevalence of CR-tuned tectal cells. In the tectal cell body region, two DS subtypes were identified with opposite preferred directions in the horizontal axis (Gabriel et al., 2012). Except for potential mutual inhibition under competing stimuli (Mysore and Knudsen, 2012, 2013), SINs may provide null direction inhibition observed in the CR-tuned DS type. This form of feedforward null-direction inhibition could contribute to fine-tuning the turning angle of an orienting swim. Collectively, these distinct cell types tuned to directional motion endow the tectum and the superior colliculus the functional role in directing eye-head-body movements toward or away from a moving object (Gandhi and Katnani, 2011).

PVNs were reported to show negative spatial summation, wherein neurons have RF size larger than their preferred size (Niell and Smith, 2005). Comparing the tuning curves of SINs and PVNs, it’s probable that large size tuned SINs provide local inhibition that underlies the negative spatial summation in PVNs. Similar to SINs, somatostatin-positive inhibitory neurons (SOMs) in the superficial layers of the mouse visual cortex exhibit increasing responses with stimulation of the RF surround and contribute to pyramidal cells’ surround suppression (Adesnik et al., 2012; Barker and Baier, 2013). Distinct size discrimination properties therefore comprise a functional module within the tectum that distinguishes differentially sized objects with ethological meanings.

If one neuron is sensitive to motion, then it’s intriguing to know whether it keeps the motion information or only show transient response profile. Our finding that SINs are motion sensitive and show sustained response provides a substrate for maintaining local motion information and allowing the tectum to assemble a representation of the overall pattern of motion in the environment for execution of distinct, ethologically relevant behaviors (Silies et al., 2014). Correspondingly, functional imaging showed that tectal neurons of larval zebrafish responded robustly to a paramecium when it started swimming but not staying still in the visual field (Muto et al., 2013).

Ablation of SINs impaired zebrafish visually guided prey capture behavior but not global looming evoked fast escape, indicating the important role of localization of fine local objects by SINs and possible mechanism of compensation or redundancy for representation of global motion by the tectal circuit. To be mentioned, our ablation manipulation was based on the Gal4 enhancer trap line which labels only a fraction of SINs. Thus we do not exclude the possibility that the whole SIN population may also play a role in fast escape evoked by looming stimuli (Dunn et al., 2016) or affect avoidance response to a moving dot a little bit larger than the size of a paramecium, which could evoke avoidance behavior (Bianco et al., 2011; Trivedi and Bollmann, 2013).

Sensory processing is strongly influenced by brain state, in which starvation positively or negatively modulates neural activities in olfactory, gustatory and visual systems across animal species according to feeding requirement and limited energy allocation (de Araujo et al., 2006; Longden et al., 2014; Marella et al., 2012; Pager et al., 1972; Root et al., 2011). The modification of response gain of SINs in different feeding states may result from neuromodulation originated from the hypothalamic-pituitary-adrenal axis (Filosa et al., 2016) and further contribute to visual size discrimination in zebrafish under behavioral choices.

Future studies are required to probe the connectivity patterns among SINs and other cell types both at the upstream and downstream and how SINs directly involve in the local circuit. In conclusion, our results coherently demonstrate that SINs serve as motion detectors for extracting local spatial displacement feature derived from environmental moving objects, which underlies the important role of the tectum or the superior colliculus in appropriate behavioral choices among vertebrate species.

## MATERIALS AND METHODS

### Zebrafish preparation

Adult zebrafish (*Danio rerio*) were maintained in the National Zebrafish Resources of China (Shanghai, China) with an automatic fish-housing system (ESEN, China) at 28 °C following standard protocols (Mu et al., 2012; Wei et al., 2012). Electrophysiological recording and calcium imaging were performed on 7–8 days post fertilization (dpf) of larval zebrafish. All zebrafish handling procedures followed the Animal Use Committee of Institute of Neuroscience, Chinese Academy of Sciences.

### Paramecium prey capture test

Zebrafish larvae were placed in a 24-well plate individually with 1.5 mL of paramecium solution containing approximately 15 paramecia per 200 μL. Intact control, ablated fish and blank control were separated sequentially to minimize effect of paramecium concentration. After adding larvae to individual well, the 24-well plate was placed in 28 °C incubator four hour later. Thereafter, remaining paramecia were counted in 200 μL solution from the well twice under a stereomicroscope and quantified as the averaged number. All values were normalized to the average number of the well without larvae. Consumed paramecia were calculated by (100%-(normalized remaining paramecia)).

### Escape behavior test

Larval behavior was monitored with an infrared-sensitive high-speed camera at 500 Hz (Redlake Motionscope M3, US). Each larva was placed in a 3.5-cm Petri dish and allowed to freely swim in the test arena for over 15 min before experiment for adaptation. The behavior of six larvae in six individual dishes was simultaneously recorded during an experiment. Eight trials with 5 min interval were performed to calculate the probability of escape behavior for each larva.

### *In vivo* electrophysiological recording

Zebrafish larvae were first paralyzed with the neuromuscular junction blocker a-bungarotoxin (100 μg/ml, Sigma) for 10–15 min, and were then embedded in ~1.5% low melting agarose (Sigma) for mechanical fixation. The extracellular solution consisted of (in mmol/L): 134 NaCl, 2.9 KCl, 2.1 CaCl_2_, 1.2 MgCl_2_, 10 HEPES and 10 glucose (290 mOsm/L, pH = 7.8). *In vivo* whole-cell recording of SINs and PVNs were made under infra-red visual guidance through a tiny cut for breaking the skin made at the middle line. The recording pipette was pulled from borosilicate glass capillaries (BF100-58-10, WPI), had a resistance in the range of 15-20 MΩ, and was tip filled with internal solution and then backfilled with internal solution. The internal solution consisted of (in mmol/L): 110 K-gluconate, 10 KCl, 2 CaCl_2_, 2 Mg-ATP, 0.3 Na_2_-GTP, 10 HEPES, 10 EDTA and 10 phosphocreatine (280 mOsm/L, pH 7.4). Recording was made with a patch-clamp amplifier (MultiClamp 700B; Axon Instruments) and signals were filtered at 5 kHz and sampled at 10 kHz using AxoScope software 10.0 (Axon Instruments). The data were discarded if the series resistance varied by >20% during recording. All drugs were purchased from Sigma-Aldrich unless otherwise mentioned.

### Calcium imaging

For calcium imaging of SINs, we used double transgenic zebrafish Tg(Gal4-1156t,UAS:GCaMP-HS) larvae that were obtained by crossing Tg(UAS:GCaMP-HS) (gift from Dr. Koichi Kawakami) with the Gal4 enhancer trap line Tg(Gal4-1156t) (ZIRC). Confocal calcium imaging was carried out under a 40×, 0.8 NA water-immersion objective using an Olympus Fluoview 1000 confocal microscope. A recording chamber was custom built with one side enclosed by a diffusive screen. The larva was mounted dorsal side up in ~1.5% agarose on the edge of a raised platform in the imaging chamber, allowing an unobstructed view of the projected stimuli on the screen of the chamber and positioned with the contralateral eye facing the projection area, which covered a visual field of approximately 110° by 110°.

### Visual stimulation

Visual stimuli were programmed using custom-written software based on MATLAB (Mathworks) and the Psychophysics Toolbox (Brainard, 1997; Pelli, 1997) and presented with a micro-projector (ASK). A colored filter directly in front of the display was used to block green light, in order to prevent interference with the fluorescence emission of GCaMP-HS. To measure the spatial RF, a pseudo random sequence of single black squares (4 × 10°) was presented for 0.2 s with an interval of 0.2 s. To measure size tuning, the black dot of various sizes that were compensated for constant visual angles was moving at a speed of 30°/s. To measure direction selectivity, the black bar was 10° in width and moving at a speed of 30°/s, in eight directions evenly spanning 360°. To measure motion sensitivity, the black dot in 10° was moving at a speed of 30°/s, in four directions evenly spanning 360°. To measure sustained response to motion, the black dot in 10° was moving at a speed of 30°/s into the RF, rotating in a series of 5 speeds from 10 to 90°/s. To measure visual adaptation, whole-field dimming pair with a series of 5 intervals from 1 s to 9 s was presented for 0.5 s, respectively. To measure escape, looming stimulation was presented with a black circle appearing, smoothly expanding at a speed of 5.4 cm/s and finally covering the whole screen. Diming stimulation with 0.4 s duration was presented. All stimuli were presented on white background.

### Data analysis

We quantified the spatial structure of neuronal RFs by using the average response in a manually selected window, which includes the largest variance, from the stimulation period. The 2D grid of neuronal membrane potentials was analyzed parametrically by fitting the response profile with a 2D Gaussian model (Womelsdorf et al., 2008). The PD and DSI were determined as the direction and magnitude of the vector sum of the averaged peak responses from three stimulus repetitions for each direction (Gabriel et al., 2012).

### Statistics

For independent data sets with only two groups we used the Student’s *t*-test or Wilcoxon rank-sum test, and for data from multiple groups we used the two-way ANOVA with mixed design or repeated measures to look for differences between factors. In data obtained from the same group under different conditions, the Paired-samples *t*-test or Wilcoxon signed-rank test was used. Differences were regarded as statistically significant at *P*-values of <0.05. All results are represented as mean ± SEM.

### Data availability

All data included in this study are available upon reasonable request from the corresponding author.

## Electronic supplementary material

Below is the link to the electronic supplementary material.

Supplementary material 1 (PDF 551 kb)
