# Supplementary material for: Optic tectal superficial interneurons detect motion in larval zebrafish
Source: Protein Cell. 2018 Nov 12;10(4):238–48. doi: 10.1007/s13238-018-0587-7 (PMC6418075; doi:10.1007/s13238-018-0587-7)
Supplement: Supplementary file 1 — Supplementary material 1 (PDF 551 kb) [file 13238_2018_587_MOESM1_ESM.pdf]

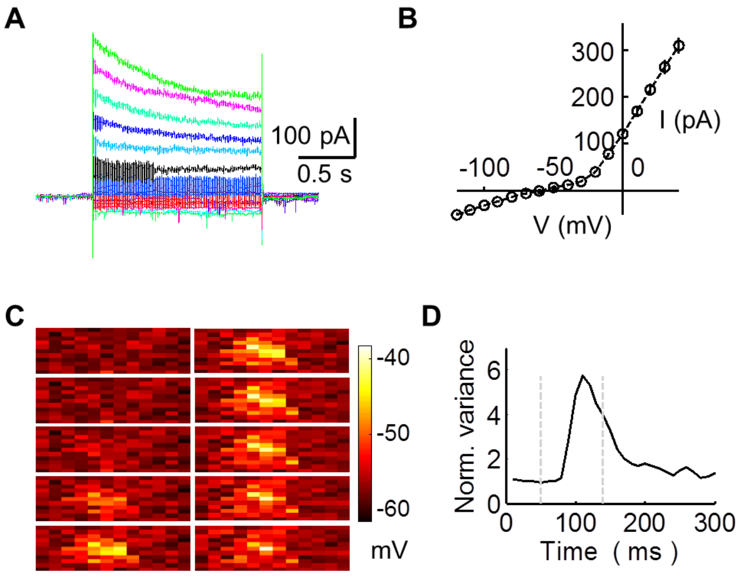

Figure S1. Voltage-current relationship and spatiotemporal map of SINs. (**A, B**) Current traces and summary of data (n = 100) for voltage-current relationship. Scale bar, 20 μm. (**C, D**) Spatiotemporal map of one SIN 50-140 ms (between gray dashed lines in (**D**)) after stimulation onset (**C**) and normalized variance of voltage responses (**D**).

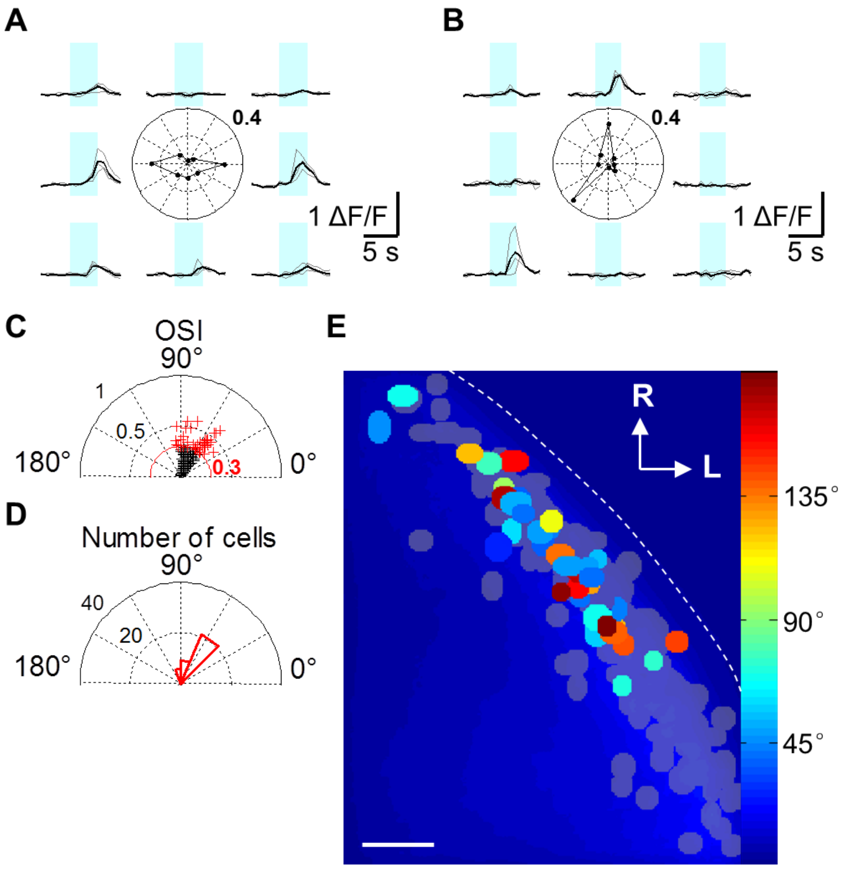

Figure S2. Orientation tuning of SINs**.** (**A, B**) Ca^{2+} transients from two somata. Center: polar plot of normalized peak amplitudes of Ca^{2+} transients. (**C**) Orientation selectivity index (OSI) and preferred orientation (PO) for all somata (n = 220). Red circle marks an OSI of 0.3, used as criterion for OS. (**D**) Histogram of POs for all OS cells from C in red. (**E**) All responsive somata from grouped experiments, color-coded according to PO. Gray somata showing responsive but not OS cells. Scale bar, 20 µm.

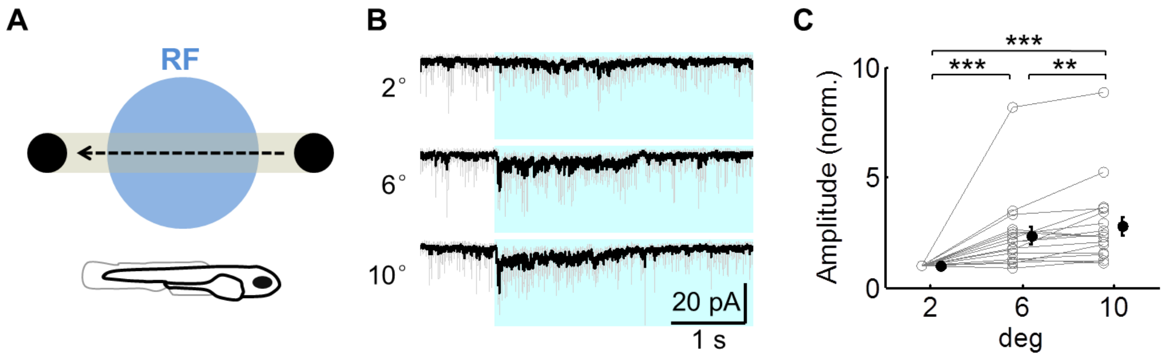

Figure S3. Size tuning of SINs. (**A**) Schematic showing moving dot stimulation. (**B**) Current traces showing responses to moving dots. (**C**) Summary of data showing

size-tuned responses to moving dots (n = 18; 2° versus 6°: p = 3.9 X 10^{-4}; 2° versus 10°: p = 2.0 X 10^{-4}; 6° versus 10°: p = 0.0050; all Wilcoxon signed-rank test).

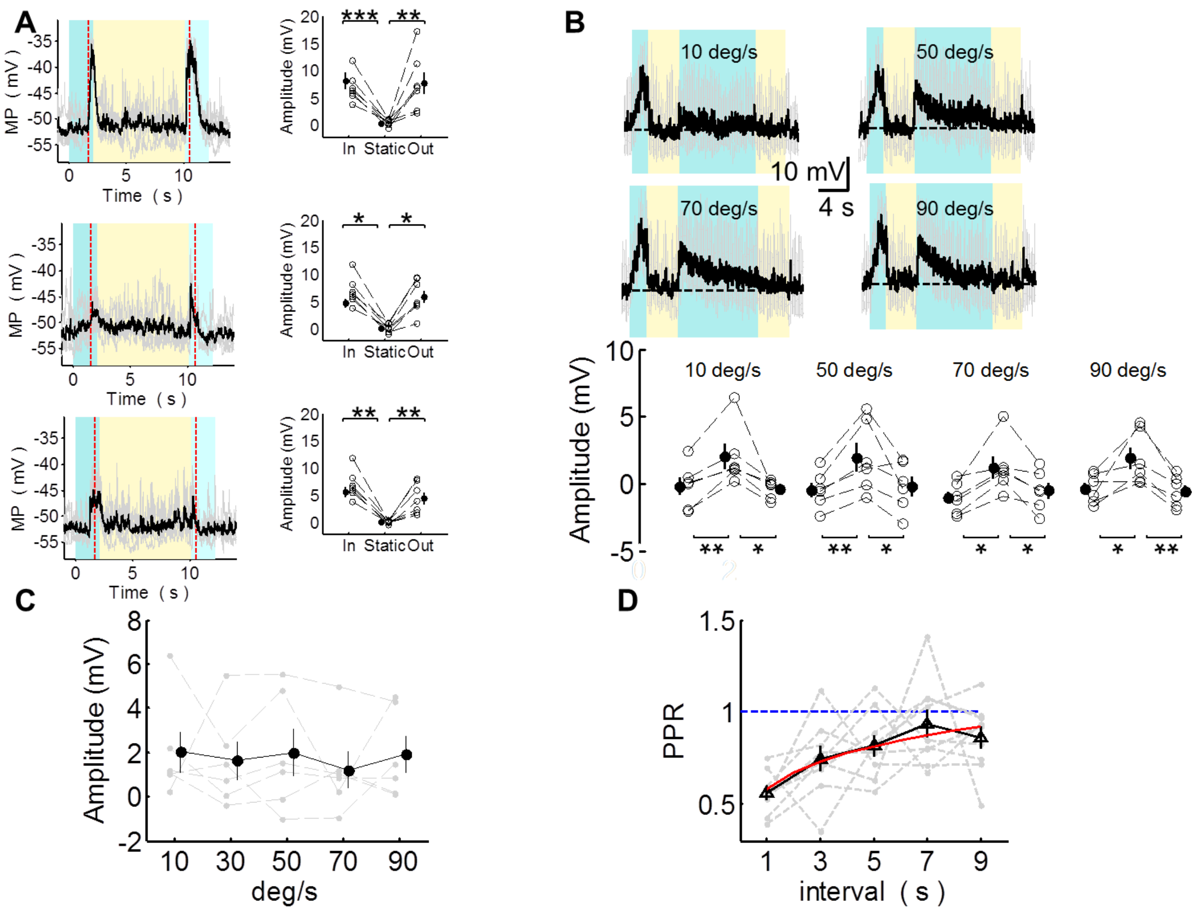

Figure S4. SINs detect motion objects. (**A**) Voltage traces (left panels) and summary of data (right panels) for responses to one dot in moving in direction of 270 (n = 7; moving in versus static: p = 7.8 X 10^{-4}; moving out versus static: p = 0.0046; Student's t-test), 0 (n = 7; moving in versus static: p = 0.0156; moving out versus static: p = 0.0156; Wilcoxon signed-rank test) and 90 (n = 7; moving in versus static: p = 0.0023; moving out versus static: p = 0.0086; Student's t-test) degree, respectively and static. (**B**) Voltage traces (top panels) and summary of data (bottom panels) for responses to one dot in moving in speed of 10 (n = 6; pre-static versus circling: p = 0.0053; circling versus post-static: p = 0.0335; Student's t-test), 50 (n = 6; pre-static versus circling: p = 0.0085; circling versus post-static: p = 0.0457; Student's t-test), 70 (n = 6; pre-static versus circling: p = 0.0313; circling versus post-static: p = 0.0313; Wilcoxon signed-rank test) and 90 (n = 6; pre-static versus circling: p = 0.0371; circling versus post-static: p = 0.0033; Student's t-test) degree/s, respectively and static. (**C**) Summary of data at various moving speeds (n = 6). (**D**) Visual adaptation for PVNs (n = 9; triangle). Red line showing power fitted curve.

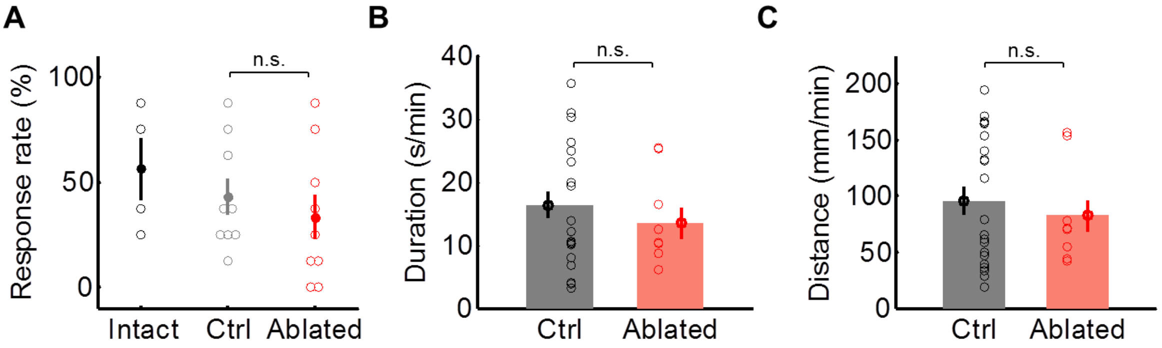

Figure S5. SIN ablation did not affect behavioral response to stationary visual stimulation and locomotion. (**A**) Diming evoked escape unaffected in SIN ablated larvae (n = 9) relative to control (n = 9, p = 0.4875, Student's t-test). Intact fish serve as control. (**B**) Duration of locomotion unaffected in SIN ablated larvae (n = 9) relative to control (n = 22, p = 0.4348, Student's t-test). (**C**) Distance of locomotion unaffected in SIN ablated larvae (n = 9) relative to control (n = 22, p = 0.9134, Wilcoxon rank-sum test).

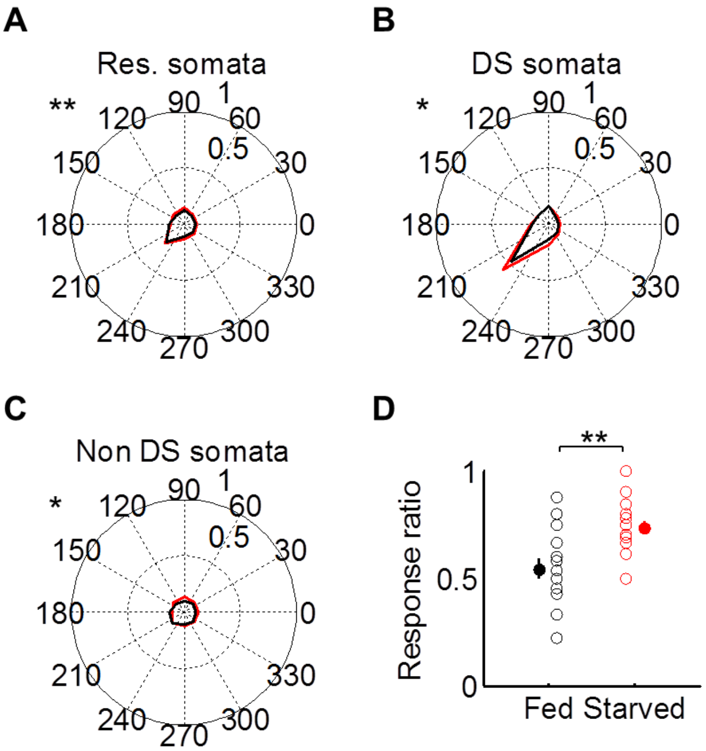

Figure S6. Gain modulation of SIN motion responses by brain state. (**A**) Summary of responses to moving bars from responsive somata between fed (n = 92/166) and starved (n = 154/214) larvae (p = 0.0156, Wilcoxon signed-rank test). (**B**) Summary of responses to moving bars from DS somata between fed (n = 23/166) and starved (n =

35/214) larvae (p = 0.0462, Wilcoxon signed-rank test). (**C**) Summary of responses to moving bars from non-DS somata between fed (n = 69/166) and starved (n = 119/214) larvae (p = 0.0437, Student’s t-test). (**D**) Summary of ratio of responsive neurons between fed (n = 15 fishes) and starved (n = 17 fishes) larvae (p = 0.0010, Student’s t-test)
